# PROVIDING ALLEGED VICTIMS AND PERPETRATORS WITH CASE MANAGEMENT TO PREVENT FUTURE CARETAKER NEGLECT

**DOI:** 10.1093/geroni/igae098.2929

**Published:** 2024-12-31

**Authors:** Samantha Tuft, Farida Ejaz, Courtney Reynolds, Jessica Bibbo

**Affiliations:** Benjamin Rose, Cleveland, Ohio, United States; Benjamin Rose Institute on Aging, Cleveland, Ohio, United States; Benjamin Rose, Cleveland, Ohio, United States; Benjamin Rose, Cleveland, Ohio, United States

## Abstract

Caretaker neglect is the second most common type of adult abuse nationwide, including Utah, where 74% of cases resulted in an ‘inconclusive’ disposition in FY 23. Practitioners from Utah Adult Protective Services (APS) are collaborating with researchers in an ongoing study to offer four months of telephone-based case management to alleged victims and perpetrators when APS closes their case as ‘inconclusive’. The goal is to prevent future reports of caretaker neglect. The study uses a four-month, pre-post design. Care coordinators interview study participants, develop and modify individualized care plans, and make referrals to services throughout the study. Preliminary data from 18 alleged victims shows their average age is 64 (range 18-59), 50% are female, and 28% live in rural areas. They experienced a significant reduction in depressive symptoms using the Patient Health Questionnaire – 2 during the study (p =.038). Data from 9 alleged perpetrators shows that their average age is 60 years (range 43-70), 78% are female, and 11% live in rural areas; their depression scores did not decline significantly, but trended downwards over time. Discussion will focus on the value of providing case management services to improve psychosocial outcomes, such as depression. Implications for practice and policy include the value of multidisciplinary collaborations to develop interventions to potentially prevent future reports of caretaker neglect, and, perhaps, other forms of abuse, neglect or exploitation.

